# Supplementary material for: zIncubascope: Long-term quantitative imaging of multi-cellular assemblies inside an incubator
Source: PLoS One. 2025 Jan 23;20(1):e0309035. doi: 10.1371/journal.pone.0309035 (PMC11756754; doi:10.1371/journal.pone.0309035)
Supplement: S1 Video — (DOCX) [file pone.0309035.s003.docx]

**S1 Video.** Timelapse of an hiPSCs cyst growing in a spherical capsule (cropped from

the full FOV) from Day 1 to Day 3.

<https://osf.io/7w9nv>
