# Supplementary material for: zIncubascope: Long-term quantitative imaging of multi-cellular assemblies inside an incubator
Source: PLoS One. 2025 Jan 23;20(1):e0309035. doi: 10.1371/journal.pone.0309035 (PMC11756754; doi:10.1371/journal.pone.0309035)
Supplement: S2 Video — (DOCX) [file pone.0309035.s004.docx]

**S2 Video.** Timelapse of hiPSCs cysts growing in spherical capsules from Day 4 to

Day 6.

<https://osf.io/cx3a2>
