# Supplementary material for: zIncubascope: Long-term quantitative imaging of multi-cellular assemblies inside an incubator
Source: PLoS One. 2025 Jan 23;20(1):e0309035. doi: 10.1371/journal.pone.0309035 (PMC11756754; doi:10.1371/journal.pone.0309035)
Supplement: S5 Video — Scale bar = 500 μm. (DOCX) [file pone.0309035.s007.docx]

**S5 Video.** Timelapse of yeast proliferating in spherical capsules over 12 hours. Scale

bar=500 µm.

<https://osf.io/v9gqu>
