# Supplementary figures and images for: zIncubascope: Long-term quantitative imaging of multi-cellular assemblies inside an incubator
Source: PLoS One. 2025 Jan 23;20(1):e0309035. doi: 10.1371/journal.pone.0309035 (PMC11756754; doi:10.1371/journal.pone.0309035)

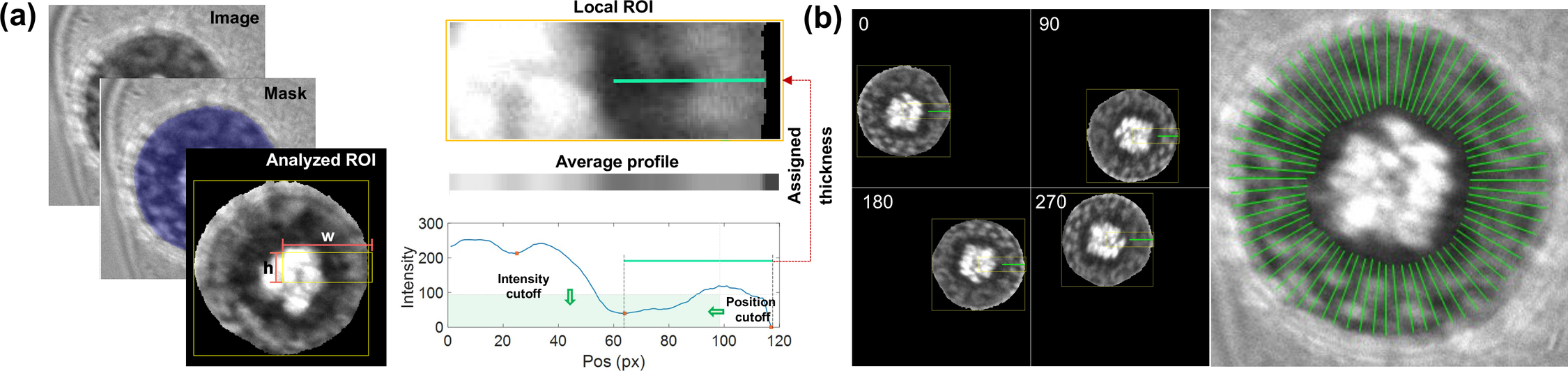

Supplement: S1 Fig — (TIF) [file pone.0309035.s008.tif]

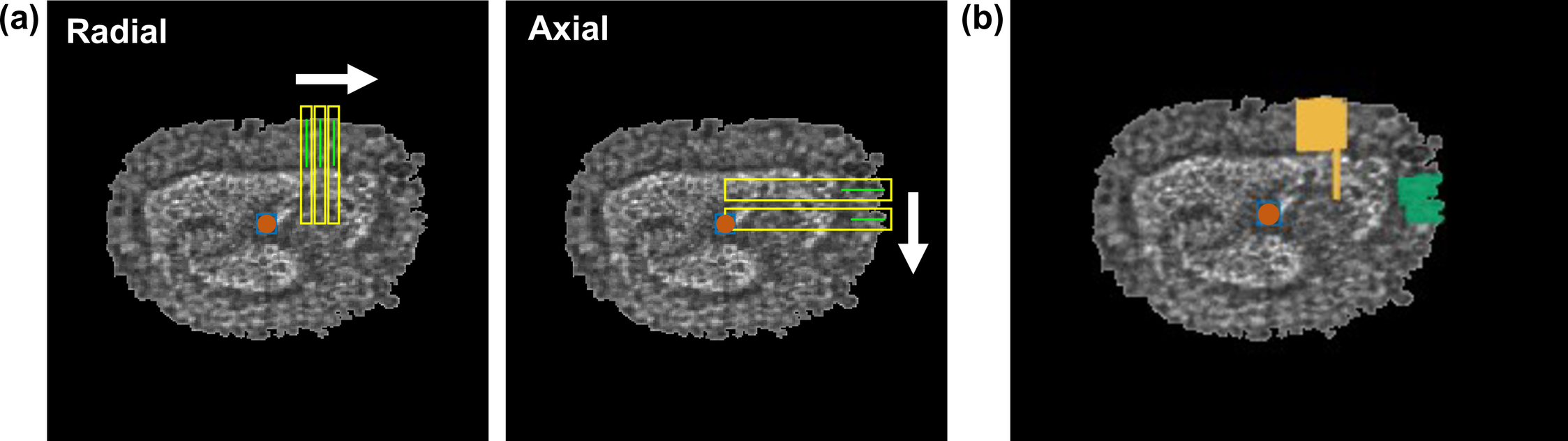

Supplement: S2 Fig — (TIF) [file pone.0309035.s009.tif]

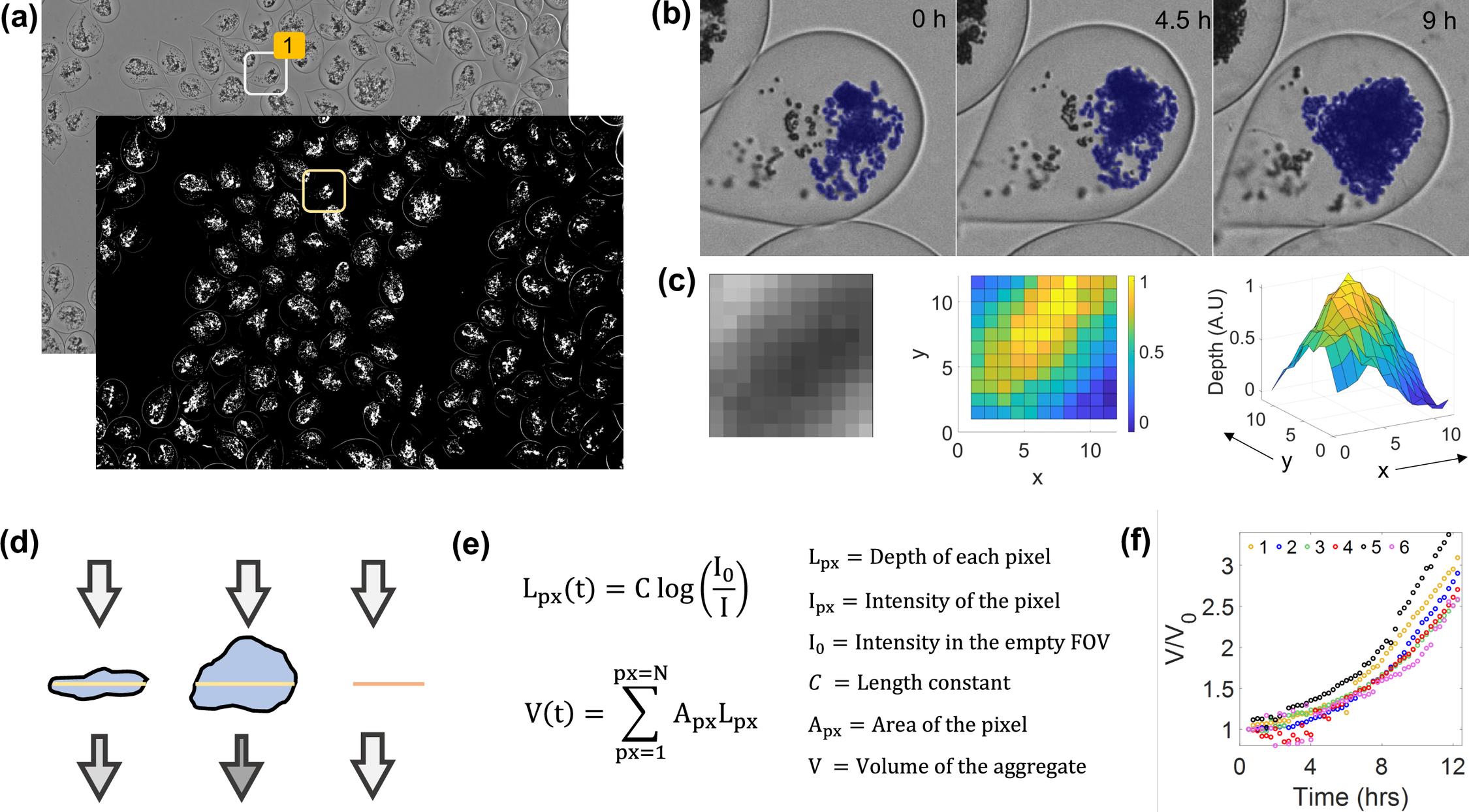

Supplement: S3 Fig — (TIF) [file pone.0309035.s010.tif]

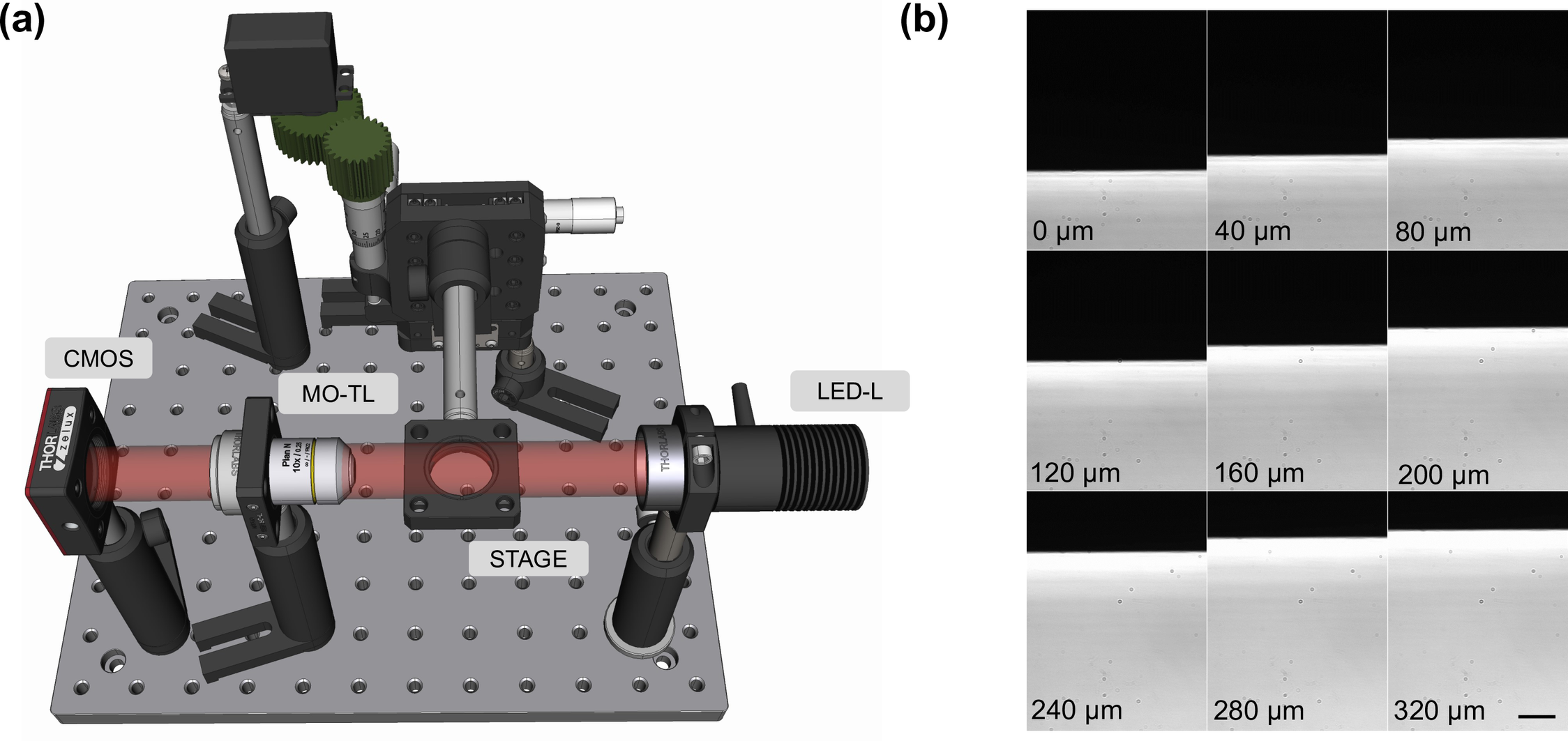

Supplement: S4 Fig — (TIF) [file pone.0309035.s011.tif]

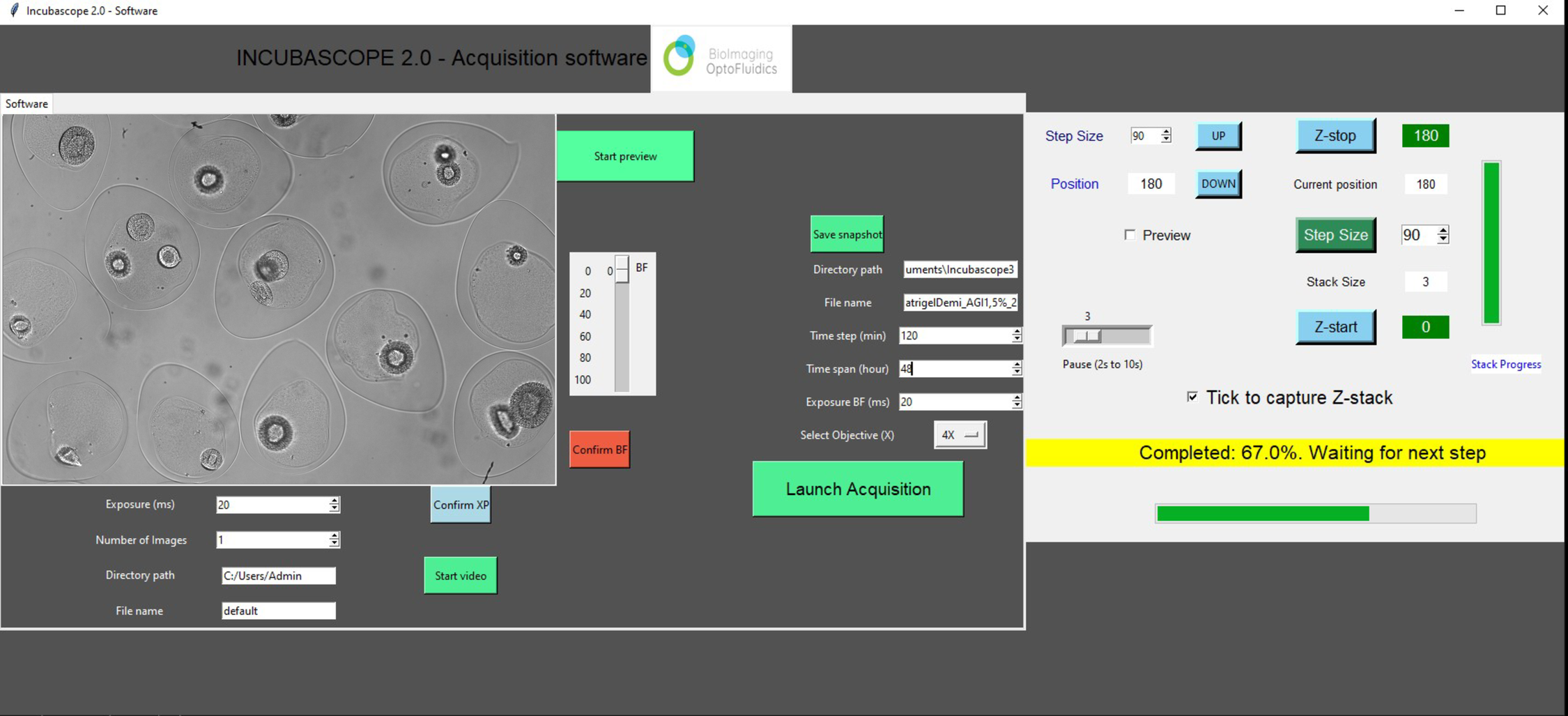

Supplement: S5 Fig — (TIF) [file pone.0309035.s012.tif]

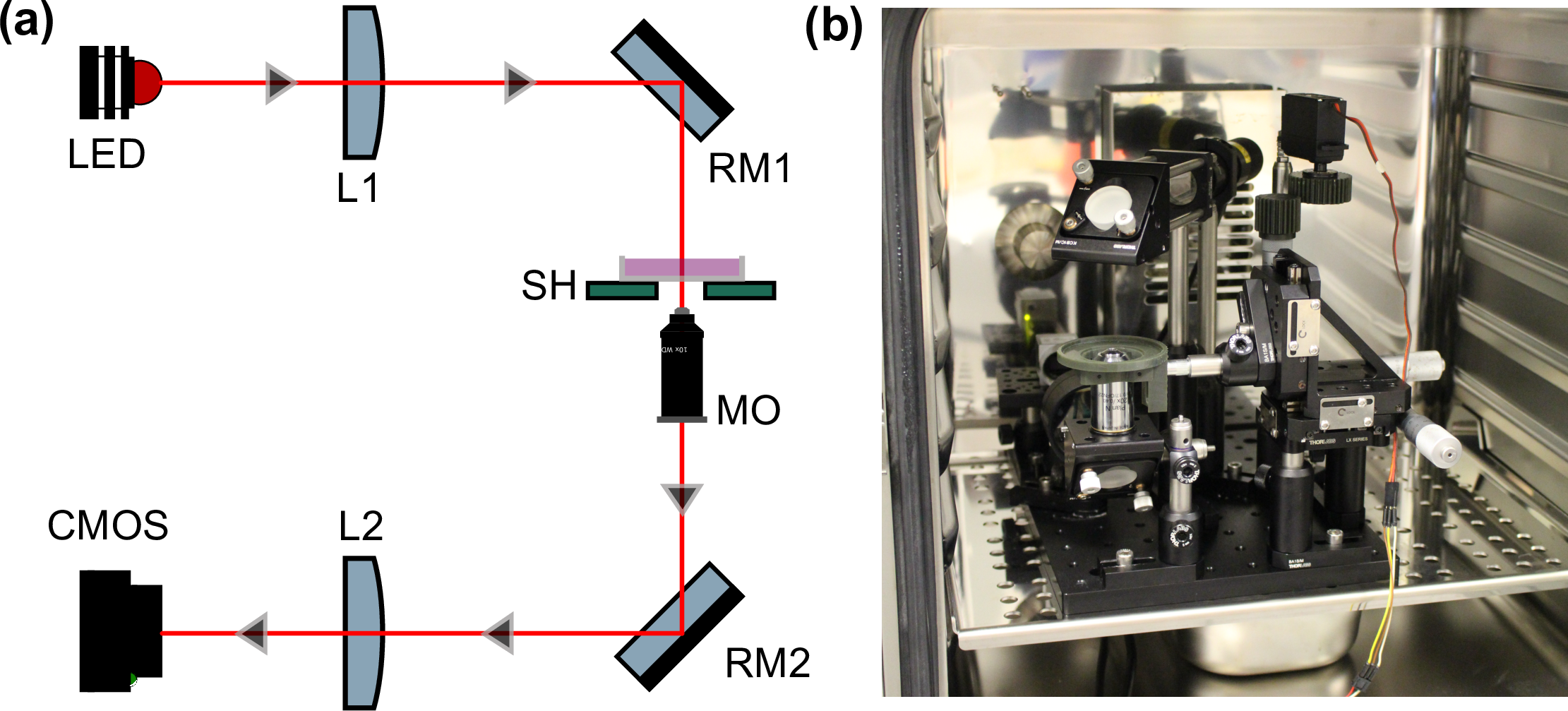

Supplement: S6 Fig — (TIF) [file pone.0309035.s013.tif]
