# Supplementary material for: zIncubascope: Long-term quantitative imaging of multi-cellular assemblies inside an incubator
Source: PLoS One. 2025 Jan 23;20(1):e0309035. doi: 10.1371/journal.pone.0309035 (PMC11756754; doi:10.1371/journal.pone.0309035)
Supplement: Attachment — Submitted filename: Response_zIncubascope_final.docx [file pone.0309035.s014.docx]

Dr Amaury Badon
LP2N, Laboratoire Photonique Numérique et Nanosciences,
Univ. Bordeaux, F-33400 Talence, France
e-mail: amaury.badon@institutoptique.fr

Bordeaux, July 16^th^, 2024

Subject: Revised version of manuscript PONE-D-24-10924

Dear editor,

Please find enclosed a revised version of our manuscript PONE-D-24-10924 that we have modified following the referee’s comments. We believe we have addressed all the comments raised. These comments were positive on our work and helped us to improve the quality of our article.

We also provide detailed answers to each point raised by the referee.

Based on the positive reviews and the answers that we have provided, we hope that our manuscript is now ready for publication in *PLOS ONE*.

Sincerely,

Amaury Badon, on behalf of all the authors

Review Comments to the Author

We thank the referee for her/his positive comments. We provide detailed answers regarding these comments below.

Reviewer #1: The manuscript presents an open-source and low-budget compact bright-field microscope built with off-the-shelf parts, which easily fits in a standard cell culture incubator and is suitable for several days-long time lapse recording of live specimens. The authors describe the setup and subsequently showcase applications with encapsulated cells, cysts, and yeast.
The element of novelty appears to be not really high, as the authors already published a very similar setup. The major improvement in this work is the integration of a motorized z-axis to record image stacks in bright field. I would suggest to consider the publication of the manuscript upon the accomplishment of major changes.
The positive aspects of this work are:
- Compact system built with off-the-shelf parts, it could be assembled and tested in few days provided that all the needed information for part purchase and assembly is given (which is not the case in the present version).
- Apparently stable over time and able to record sufficiently long time-lapse data in bright-field.
- Any open-source, low-cost solution to perform basic time-lapse live imaging experiments with bright-field illumination is positive for the scientific community, especially for low-budget laboratories.

Criticisms/recommendations
- With one microscope only one condition can be examined. This limits its applicability to specific cases, so it’s not as universal as claimed and the authors should discuss it more critically.

We are not sure to fully understand the comment of the referee. In this article, we do not claim to present an universal imaging platform. We provide a simple and general one illustrated by imaging very different biological samples (human induced pluripotent stem cells in spherical capsules, in tubular capsules and yeast). As the field of view can be relatively large, one can divide it into different conditions by using compartmentalized Petri dishes for instance. Another solution is to have several zIncubascopes and to perform acquisitions in parallel. We now stress on this point in the main text :

“In this new configuration, two zIncubascopes can even be housed inside standard incubators, thus increasing the capability of imaging either more numerous, more diverse samples or different experimental conditions.”

- A part list for the building of the microscope is missing. A person skilled in opto-mechanics and microscopy will be able to identify the off-the-shelf parts immediately. However, users with less experience in these fields, who are an important target of the paper, will find very useful a list of parts with catalogue number and price, and the authors should add it.

We thank the reviewer for this advice and we have updated our repository with a detailed list of parts with catalog number and an estimation of the price. We now mention this in the supplementary information file (SI, section 5) :

“Note that we provide here [6] a list of the part required to build the setup accompanied by instructions on how to assemble it.”

- The GitHub repository contains the code, CAD data, and experimental data (e.g., time-lapse data). However, there is no explanation whatsoever, tips and tricks, troubleshooting, etc. Inserting these details in the repository will make the adoption of the system, particularly by inexperienced users, much easier. I suggest to revise the repository adding a comprehensive explanation to accompany the material. Without this, the repository is not useful.

We thank the reviewer for this constructive comment. In a new document added onto our repository (zIncunascope_instructions.pdf), we now provide detailed instructions on how to assemble the apparatus but also on how to install the code and use it to perform acquisitions. We provide illustrations and screen captures to ensure a clear understanding of the different steps. In addition, we also added comments in our codes to clearly explain how to run the codes, both in Matlab and Python. We believe these efforts will facilitate adoption by other users.

- It would be recommendable to give hints on how to upgrade the system, e.g., with a motorized stage. In fact, a motorized xy-stage would be important for the community of users, and make the device more „universal “. The authors may want to comment on this.

We thank the referee for this excellent suggestion. Our main motivation was to develop a simple and cost effective microscope. For this, we preferred to have a relatively large field of view and to do without any motorized stage. We only developed a custom solution for the axial direction in order to capture more information on 3D specimens. Of course, having a motorized stage in XY would be a great improvement and to do so we could :

(1) Implement the same strategy as we did for the Z position. Similar gear mounts coupled with motors can be installed on the X and Y micrometer heads to achieve displacements in the XY plane. It is directly compatible with our apparatus in terms of space and also electronics since the Arduino board has several free remaining pins.
(2) Install a commercial XY or XYZ stage. Of course this solution will provide better performances but the price of such a stage is of the order of the total price of our apparatus (4 k€). Additionally, we do not know how the conditions inside an incubator will affect the performance and durability of a commercial stage.

We now mention these two strategies in the discussion section :
“If needed, our system can also be easily upgraded with a motorized stage to increase
the FOV. The solution we provide for the motor driven z-stack capabilities can be
extended in the 3 dimensions. This approach is both ergonomic and cost-effective.
Alternatively, compact commercial motorized stages that provide better performances are often compatible with a Python control but the price of such a system is usually more
expensive than the total price of the zIncubascope.”

- The system seems to be quite incremental with respect to the previous publication by the same authors. All the professional objective lenses have the same parfocal length and tube lens distance, so the previous system could already be used with other objective lenses, I suppose. The authors should clarify this point.

The tube lens distances can be quite different from a manufacturer to another one (from 165 mm with Zeiss, to 200 mm with Nikon). Parfocal length can also vary depending on the manufacturer and the application. For instance, in our previous publication, we were employing a microscope objective with a working distance of 5 cm and a parfocal length of 95 mm. In this work, we focus on objectives with a “conventional” parfocal length of 45 mm. This replacement modifies the setup drastically in terms of assembly and compactness of the apparatus. . In addition, in this work we stress that our imaging platform is composed of the apparatus and an analysis pipeline. The modification on the setup (motorized axial position, system more compact, compatible with 45 mm parfocal length objectives) is supported by the development of open-source analysis codes that will help to extract quantitative information captured with the zIncubascope. Here, we showed that the combination of these two aspects can lead to very precise insights on complex biological systems. This is clearly stated in the introduction of our article (line 63-69).

Journal Requirements:

2. Thank you for stating the following financial disclosure: 
"This project was supported by grants from the French National Agency for Research (ANR-22-CE42-0019, ANR-21-CE18-0038, ANR-21-CE19-0029) and the Institut National du Cancer ( PLBIO 20-135 ). The authors also acknowledge the financial support from the Grand Research Program LIGHT Idex University of Bordeaux, the Graduate program EUR Light S & T PIA3 ANR-17-EURE-0027 and GdR ImaBio."

Please state what role the funders took in the study.  If the funders had no role, please state: ""The funders had no role in study design, data collection and analysis, decision to publish, or preparation of the manuscript."" 
If this statement is not correct you must amend it as needed. 
Please include this amended Role of Funder statement in your cover letter; we will change the online submission form on your behalf.

Indeed, the funders took no role in the study. Thanks to add the online submission form with the following sentence : "The funders had no role in study design, data collection and analysis, decision to publish, or preparation of the manuscript.""

3. Thank you for stating the following in the Acknowledgments Section of your manuscript: 
"We thank all the other members of the BiOf (Bioimaging and Optofluidic) team, in particular Camille Douillet, Aur´elien Richard, Laetitia Andrique, L´eon Rembotte, Fernanda Lopez-Garcia, for beta testing the device and suggesting improvements. We are also grateful to all the colleagues that encouraged us to develop a user-friendly version of the zIncubascope by expressing enthusiastically their need for such an instrument. This project was supported by grants from the French National Agency for Research (ANR-22-CE42-0019, ANR-21-CE18-0038, ANR-21-CE19-0029) and the Institut National du Cancer ( PLBIO 20-135 ). The authors also acknowledge the financial support from the Grand Research Program LIGHT Idex University of Bordeaux, the Graduate program EUR Light S & T PIA3 ANR-17-EURE-0027 and GdR ImaBio."

We note that you have provided funding information that is not currently declared in your Funding Statement. However, funding information should not appear in the Acknowledgments section or other areas of your manuscript. We will only publish funding information present in the Funding Statement section of the online submission form. 
Please remove any funding-related text from the manuscript and let us know how you would like to update your Funding Statement. Currently, your Funding Statement reads as follows: 
"This project was supported by grants from the French National Agency for Research (ANR-22-CE42-0019, ANR-21-CE18-0038, ANR-21-CE19-0029) and the Institut National du Cancer ( PLBIO 20-135 ). The authors also acknowledge the financial support from the Grand Research Program LIGHT Idex University of Bordeaux, the Graduate program EUR Light S & T PIA3 ANR-17-EURE-0027 and GdR ImaBio."
Please include your amended statements within your cover letter; we will change the online submission form on your behalf.

In the new version of the manuscript, we removed the funding related text form. The current funding statement is correct :
"This project was supported by grants from the French National Agency for Research (ANR-22-CE42-0019, ANR-21-CE18-0038, ANR-21-CE19-0029) and the Institut National du Cancer ( PLBIO 20-135 ). The authors also acknowledge the financial support from the Grand Research Program LIGHT Idex University of Bordeaux, the Graduate program EUR Light S & T PIA3 ANR-17-EURE-0027 and GdR ImaBio."

4. Please upload a copy of Supporting Information Figure/Table/etc. S1-S5 Video which you refer to in your text on page 14/17.

All the videos are uploaded in a project on the Open Science Framework (OSF) website. Here is the address of the repository : <https://osf.io/q6npr/>.
URL links are provided in the online submission form.
